# Human AP endonuclease inefficiently removes abasic sites within G4 structures compared to duplex DNA

**DOI:** 10.1093/nar/gku417

**Published:** 2014-05-21

**Authors:** Christopher Broxson, Jaclyn N. Hayner, Joshua Beckett, Linda B. Bloom, Silvia Tornaletti

**Affiliations:** 1 Departments of Anatomy and Cell Biology,; 2 Biochemistry and Molecular Biology,; 3 Medicine, University of Florida College of Medicine, 1600 SW Archer Road, Gainesville, FL 32610, USA

## Abstract

Excision repair processes are essential to maintain genome stability. A decrease in efficiency and fidelity of these pathways at regions of the genome that can assume non-canonical DNA structures has been proposed as a possible mechanism to explain the increased mutagenesis and consequent diseased state frequently associated with these sites. Here we describe the development of a FRET-based approach to monitor the presence of G quadruplex (G4) DNA, a non-canonical DNA structure formed in runs of guanines, in damage-containing single-stranded and double-stranded DNA. Using this approach, we directly show for the first time that the presence within the G4 structure of an abasic site, the most common lesion spontaneously generated during cellular metabolism, decreases the efficiency of human AP endonuclease activity and that this effect is mostly the result of a decreased enzymatic activity and not of decreased binding of the enzyme to the damaged site. This approach can be generally applied to dissecting the biochemistry of DNA repair at non-canonical DNA structures.

## INTRODUCTION

DNA structures folded into the non-canonical G quadruplex (G4) conformation have been recently proposed to participate in vital cellular processes including initiation of DNA replication, regulation of gene expression and maintenance of chromosome ends (1–5). G4 DNA also localizes to regions of the genome characterized by genetic instability, suggesting that G4 formation may impair recognition and processing by nucleic acid directed proteins and enzymes eventually leading to mutagenesis (6–8). Excision DNA repair enzymes are essential to maintain genome stability by removing deleterious DNA lesions that would otherwise result in mutations, leading to human disease (9,10). Little is known about the efficiency and fidelity of excision repair pathways at genomic regions that can assume non-canonical DNA structures. Increased exposure to mutagenic agents at sites of unusual structures combined with impaired recognition and repair of DNA damage by the DNA repair machinery at these sites may render these regions particularly susceptible to mutations. Several genetic studies have shown that excision repair processes are implicated in the mutagenesis associated with regions that fold into non-B structures (7,11–19). Furthermore, transcription through these regions plays a central role in promoting formation of these non-canonical structures and in the associated mutagenesis (7,20–22). However, due to the lack of effective methods to detect G4 DNA formation in damage-containing DNA *in vitro*, the molecular events that are responsible for genetic changes remain to be investigated.

**Figure 1.**
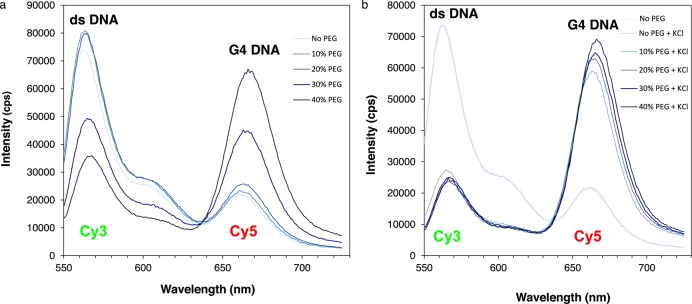
DNA sequences used in this study. (**A**) Sequence of the Nuclease Hypersensitive element III_1_ from the c-myc gene. Bases are numbered according to the sequence of NHEIII_1_. Cy3 and Cy5 fluorophores were incorporated at positions T10 and T28, for FRET analyses, marked in green and red, respectively. Two G4 folding patterns of NHEIII_1_, myc1245 and myc2345 are shown. Gs participating in G quartet formation are indicated as green circles. (**B**) Dimethyl sulphate (DMS) footprinting experiment showing G4 formation in ds c-myc-WT. DNA substrates for DMS footprinting consisted of ds c-myc-WT labeled at the 5' end of the G4 containing strand with ^32^P. The complementary strands were annealed by incubation from 95°C to 37°C in the presence or absence of 100 mM KCl and 20% PEG 200 to generate DNA oligonucleotides in which the G4-forming sequence was folded (lane 2) or not (lane 1) into G4 structure. G residues protected from DMS methylation are marked with a light gray circle, those that are not protected are marked with a filled circle, those that are partially protected are marked with a dark gray circle.

We have recently developed novel approaches to promote the transition to G4 DNA under conditions that mimic those found in cells (23,24). In addition, we have shown that the presence of the most frequent spontaneous DNA lesions, abasic site and 8-oxoguanine within the G4 -forming sequence modulates the structural transition to G4 DNA in the nuclear hypersensitive element III_1_ (NHEIII_1_) from the c-myc gene (24). This G4-forming sequence is composed of a polypurine/polypyrimidine tract that can transition from duplex to quadruplex DNA structure *in vivo* (25) and has been implicated in c-myc gene regulation in normal and cancer cells (Figure 1A).Given that this G4-forming sequence localizes near the most frequent translocation hot spot for the c-myc gene in B-cell malignancies, it is particularly relevant for studying mechanisms of mutagenesis (26–28).

Here we describe the development of a fluorescence resonance energy transfer (FRET)-based approach to monitor the presence of G4 DNA in damage-containing single-stranded and double-stranded DNA. We utilize this method to detect for the first time G4 DNA in the double-stranded c-myc NHEIII_1_ repeat when an abasic site is located within the repeat. Using this substrate, we found that the presence of the AP site within the G4 structure decreases the efficiency of human Apurinic/apyrimidinic (AP) endonuclease (hAPE1) activity and that this effect is mostly due to a decreased enzymatic activity and not of decreased binding of the enzyme to the damaged site. The combined use of the methodologies developed in this study will open new opportunities for biochemical characterization of DNA repair at non-canonical DNA structures.

## MATERIALS AND METHODS

### Proteins and reagents

T7 RNA polymerase (T7 RNAP) was purchased from Promega. Proteinase K was from Invitrogen. Restriction enzymes and hAPE1 were from New England Biolabs. APE1-D210A was prepared as described previously (29). DNA oligonucleotides were purchased from IDT and Midland. Highly purified nucleoside triphosphates (NTPs) were purchased from Amersham Pharmacia Biotech. Radiolabeled nucleotides were from MP Biomedical. Piperidine and dimethyl sulphate (DMS) were from SIGMA. Polyethylene glycol (PEG) 200 was from Fluka.

### Preparation of G4 DNA-containing templates

Synthetic single-stranded DNA templates were prepared as previously described (24). The oligomer sequences are listed in Table 1. Synthetic double-stranded DNA templates were obtained by incubating 10 pmoles of each strand in a final volume of 10 μl for 3 min at 85°C, followed by slow cooling down from 85°C to 37°C. To induce G4 DNA formation, the annealing reaction was carried out in the presence of 100 mM KCl and various concentrations of PEG 200 as indicated in the text. Control samples consisted of double-stranded substrates in which PEG or KCl or both were omitted from the annealing reaction. Control samples in which KCl was substituted with LiCl were also included. After the annealing step, samples were stored at −20°C.

**Table 1.** Sequences used in this study (5'–3')

T7 primer
5'-TAATACGACTCACTATA-3'
c-myc-WT
5'-gctacatgctctagatct/ggaaggggtgggaggggtgggaggggt/CTG
CACccagctgcttcgtccgaagaccc*tatagtgagtcgtatta*-3'
c-myc-A12
5'-gctacatgctctagatct/ggaaggggtgggaggAgtgggaggggt/CTG
CACccagctgcttcgtccgaagaccc*tatagtgagtcgtatta*-3'
c-myc-AP12
5'-gctacatgctctagatct/ggaaggggtgggaggXapgtgggaggggt/CTG
CACccagctgcttcgtccgaagaccc*tatagtgagtcgtatta*-3'

Sequences delimited by (/) correspond to wild type (WT) and mutant sequences from the c-myc NHEIII_1_; sequences shown in italic correspond to the T7 RNA polymerase promoter; Xap, apurinic (abasic) site.

### Dimethyl sulphate footprinting assays

2.5 pmoles of 91 mers containing the c-myc G repeat were end-labeled with [γ-^32^P] ATP using polynucleotide kinase. These oligonucleotides were annealed to the complementary strand by heat denaturation at 85°C followed by slow cooling down to 37°C in the presence or absence of 100 mM KCl and 20% PEG 200. The 91 mers were cooled down to 4°C before being added to a solution containing 50 mM sodium cacodylate pH 7.0, 1 mM EDTA pH 8.0. 5 μl of DMS were left to react for 5 min at room temperature. The reactions were stopped by addition of 1.5 M sodium acetate, pH 7.0, 1 M β-mercaptoethanol, 1 μg/ml tRNA. DNA was ethanol precipitated and resuspended in 1 M piperidine, freshly diluted in water. After cleavage at 90°C for 30 min, reactions were stopped by chilling in ice followed by ethanol precipitation. The samples were resuspended in 100 μl of water and dried overnight in a centrifugal vacuum concentrator. Samples were resuspended in 4 μl of formamide dye (94% formamide, 2 mM EDTA, 0.05% bromophenol-blue, 0.05% xylene-cyanol), followed by denaturation for 3 min at 90°C. The DNA samples were separated on a 12% denaturing polyacrylamide gel in Tris borate-EDTA containing 7 M urea. Gels were dried and autoradiographed using intensifying screens.

### BsgI restriction digestions

DNA substrates labeled with ^32^P at the 5' ends (5 nM) were incubated at 37°C for 5 min in a mixture containing 50 mM potassium acetate, 20 mM Tris-acetate, 10 mM magnesium acetate, 100 μg/ml bovine serum albumin (BSA), 80 μM S-adenosylmethionine and 5 U BsgI. 10 mM KCl and 2% PEG were also included in control reaction mixtures containing duplex DNA to reproduce identical conditions as those of reactions containing the G4 substrate. The DNA samples were separated on a 12% non-denaturing polyacrylamide gel in Tris borate-EDTA. Gels were dried and autoradiographed using intensifying screens.

### Preparation of DNA templates for transcription

Synthetic DNA templates for transcription reactions with T7 RNAP consisted of DNA oligonucleotides in which a double-stranded T7 promoter region was generated by annealing a 17 mer oligonucleotide of sequence complementary to the T7 promoter sequence at the 3' end of the single-stranded oligonucleotide. The oligomer sequences are listed in Table 1. T7 transcription substrates were obtained by incubating 10 pmoles of T7 primer with 10 pmoles of 91 mer oligonucleotide in a final volume of 10 μl for 3 min at 85°C, followed by slow cooling down to 37°C. After the annealing step, samples were kept at −20°C. To induce G4 DNA formation the annealing reaction was carried out in 100 mM KCl. Control samples were incubated in 100 mM LiCl or in TE buffer.

### T7 RNAP transcription reactions

The DNA templates at a final concentration of 0.1 μM were incubated at 37°C in a mixture of 50 units of T7 RNAP, 40 mM Tris-HCl, pH 7.9, 6 mM MgCl_2_, 2 mM spermidine, 10μCi [α-^32^P] GTP, 10 mM dithiothreitol, 212 units of RNAsin, 200 μM ATP, CTP, UTP, 20 μM GTP. Incubation continued at 37°C for 30 min. Reactions were stopped by addition of 5 μg of proteinase K, 1% SDS, 100 mM TrisHCl (pH 7.5), 50 mM EDTA and 150 mM NaCl, followed by incubation for 15 min at room temperature. The nucleic acids were precipitated with ethanol, resuspended in formamide dye, and denatured at 90°C for 3 min. The transcription products were resolved on a 12% denaturing polyacrylamide gel in Tris borate-EDTA containing 7 M urea. Gels were dried and autoradiographed using intensifying screens.

### hAPE1 AP endonuclease activity assays

DNA templates consisted of double-stranded 91 mer oligonucleotides containing a single tetrahydrofuran at position 12 of the c-myc NHEIII_1_. DNA templates at a final concentration of 10 nM were incubated at 37°C in a 10 μl reaction mixture containing 20 mM Tris-acetate (pH 7.9), 50 mM potassium acetate, 10 mM magnesium acetate, 1 mM dithiothreitol and 0.6 units of hAPE1. In addition, 10 mM KCl and 1.5% PEG were also included in reaction mixtures containing duplex DNA to reproduce identical conditions as those of reactions containing the G4 substrate. Samples were collected at increasing time points as described in the legend to Figure 7. Reactions were stopped by addition of 5 μg of proteinase K, 1% SDS, 100 mM Tris HCl (pH 7.5), 50 mM EDTA and 150 mM NaCl, followed by incubation for 15 min at room temperature. DNA was precipitated with ethanol, resuspended in formamide dye and denatured at 90°C for 3 min. The reaction products were resolved on a 12% denaturing polyacrylamide gel in Tris borate-EDTA containing 7 M urea. Gels were dried and autoradiographed using intensifying screens. Products were quantified using a Typhoon phosphorimager and ImageQuant software from GE Healthcare.

**Figure 2.**
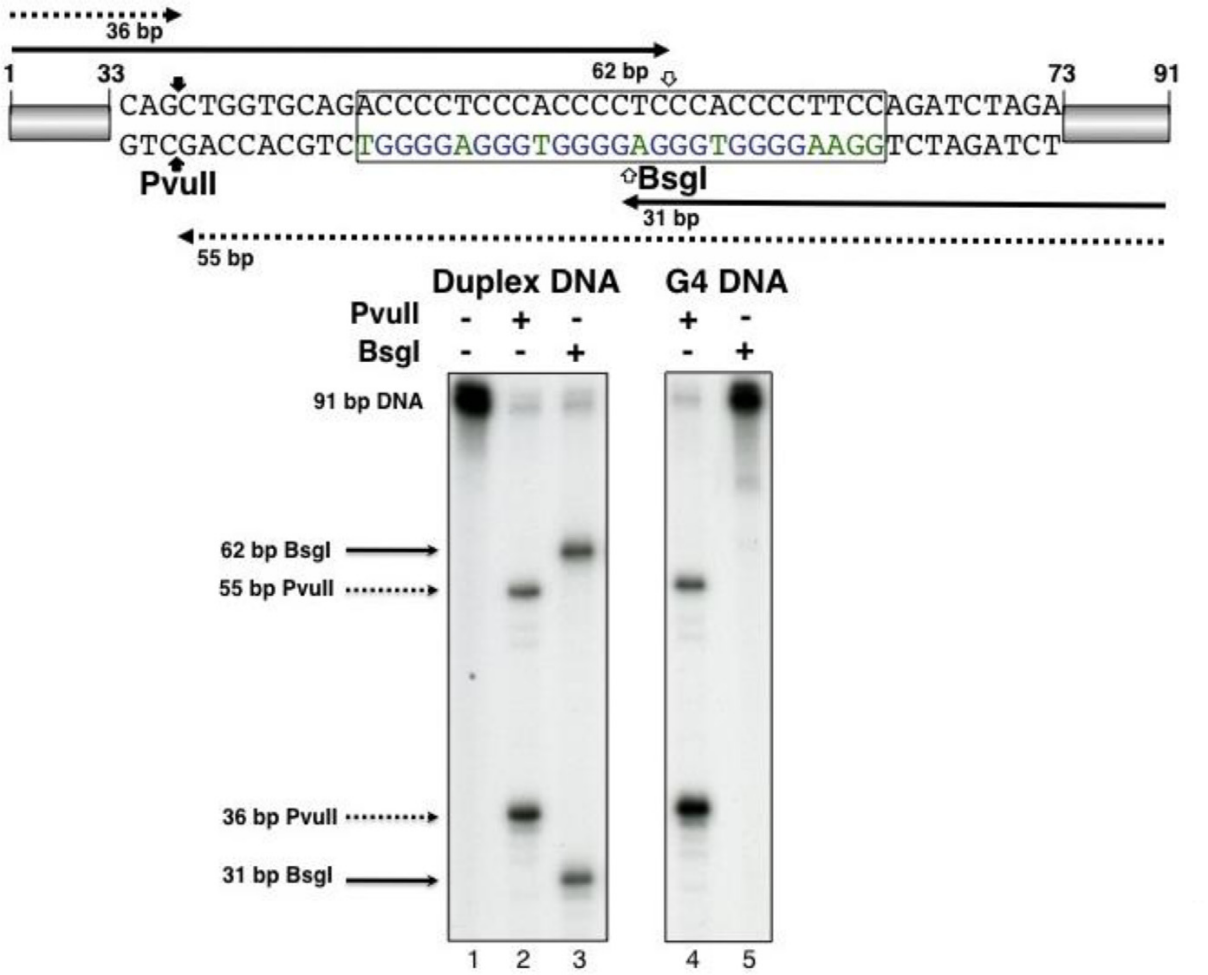
G4 DNA formation inhibits digestion by restriction enzyme BsgI. Lane 1: 91 bp c-myc-WT; lanes 2 and 4: 91 bp c-myc-WT digested with PvuII; lanes 3 and 5: 91 bp c-myc-WT digested with BsgI; ds c-myc-WT in quadruplex structure were obtained by annealing the complementary strands in the presence of KCl and PEG, as described in Materials and Methods (lanes 4 and 5). The c-myc NHEIII_1_ sequence is delimited by a box. BsgI and PvuII cleavage sites are marked with open and filled arrows, respectively. Sizes of DNA fragments expected from PvuII or BsgI digestion are marked by filled (BsgI) or dotted lines (PvuII).

**Figure 3.**
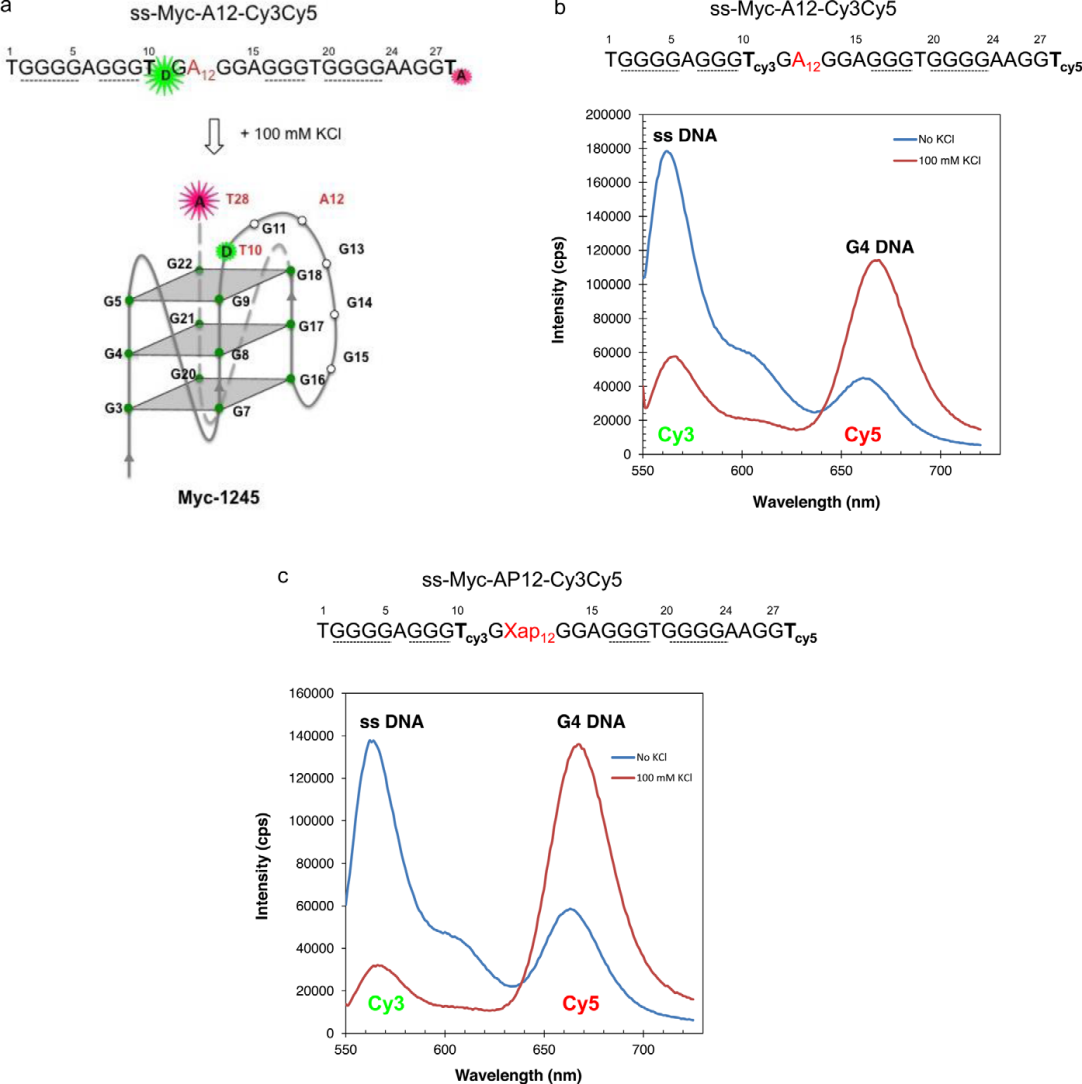
FRET assay to detect G4 DNA formation in damaged or undamaged DNA. (**a**) Scheme showing the principle of the assay. When the G4 DNA structure forms, it brings the donor Cy3 (D) and acceptor Cy5 (A) closer. Energy transfer from the donor to acceptor will quench donor fluorescence and increase acceptor fluorescence. (**b**) Emission spectra of ss-myc-A12-Cy3Cy5 pre-incubated (Red—100 mM KCl) with 100 mM KCl to induce G4 formation or not (Blue—No KCl). (**c**) Emission spectra of ss-myc-AP12-Cy3Cy5 pre-incubated (Red—100 mM KCl) with 100 mM KCl to induce G4 formation or not (Blue—No KCl).

**Figure 4.**
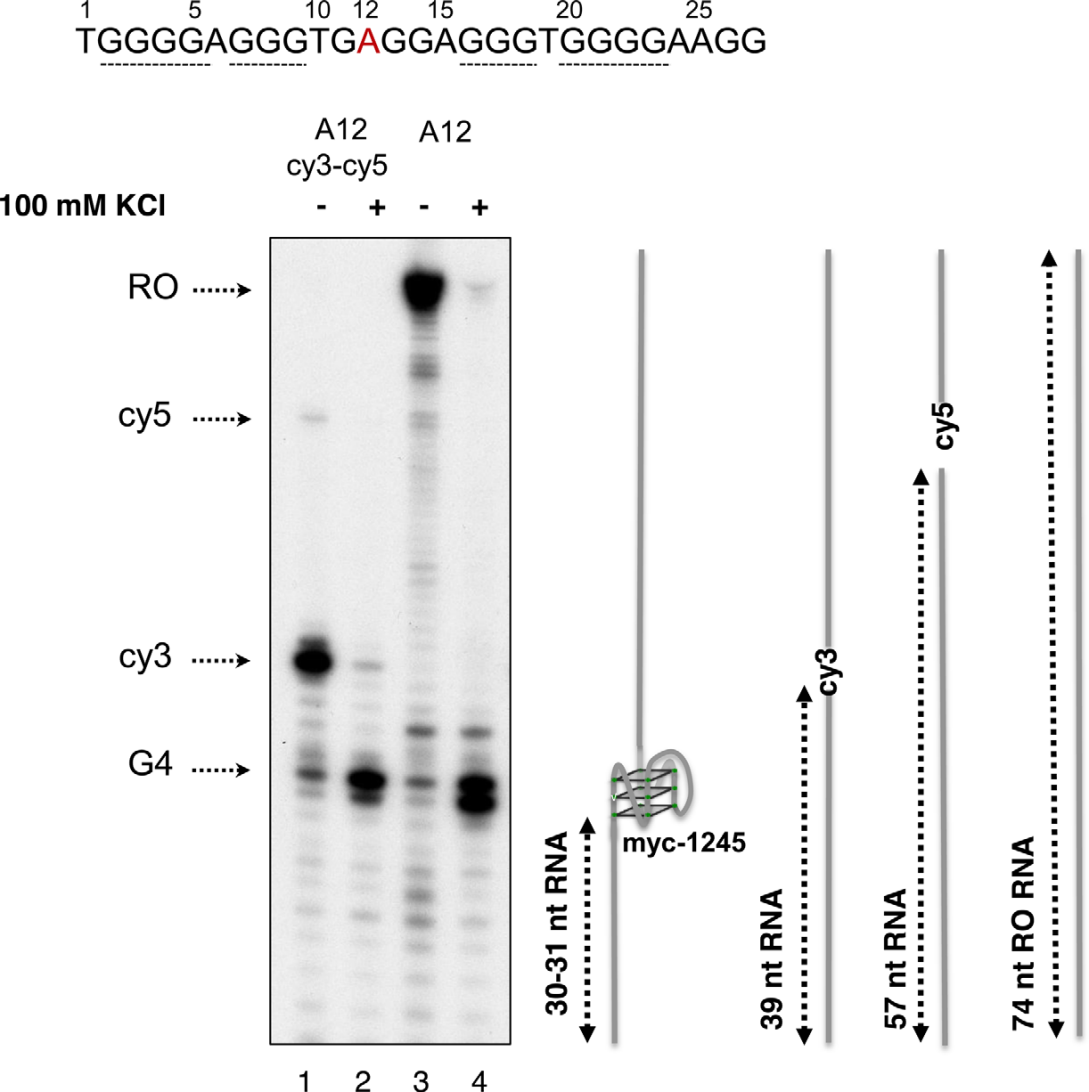
Detection of G4 DNA formation by transcription stop assay. DNA templates were transcribed *in vitro* such that the transcripts were radioactively labeled. Elongation was allowed to proceed for 30 min at 37°C after addition of NTPs to the reaction, followed by RNA purification and separation on 12% denaturing polyacrylamide gels. A schematic representation of transcription substrates c-myc-A12 and c-myc-A12-cy3-cy5 and the expected sizes of the transcripts resulting from arrest at myc1245 (30–31 nt RNA), Cy3 (39 nt RNA), Cy5 (57 nt RNA) in addition to the Runoff RNA (74 nt RO RNA) are shown to the right of the gel picture.

**Figure 5.**
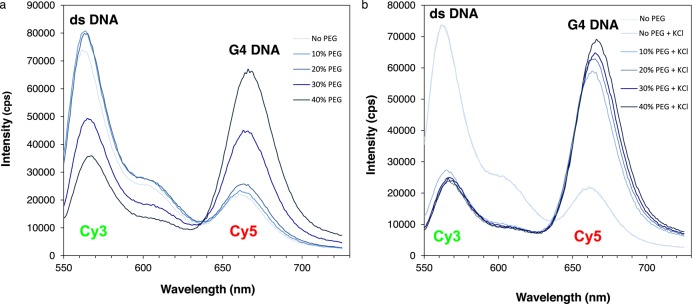
Effect of PEG concentration on G4 DNA detection in ds c-myc-A12. Emission spectra of ds-myc-A12 prepared by annealing the complementary strands in the presence of PEG 200 concentrations ranging from 0 to 40% in the absence (panel A) or presence (panel B) of 100 mM KCl to induce G4 formation. Spectra are colored such that the shade of blue becomes darker as the PEG concentration increases.

**Figure 6.**
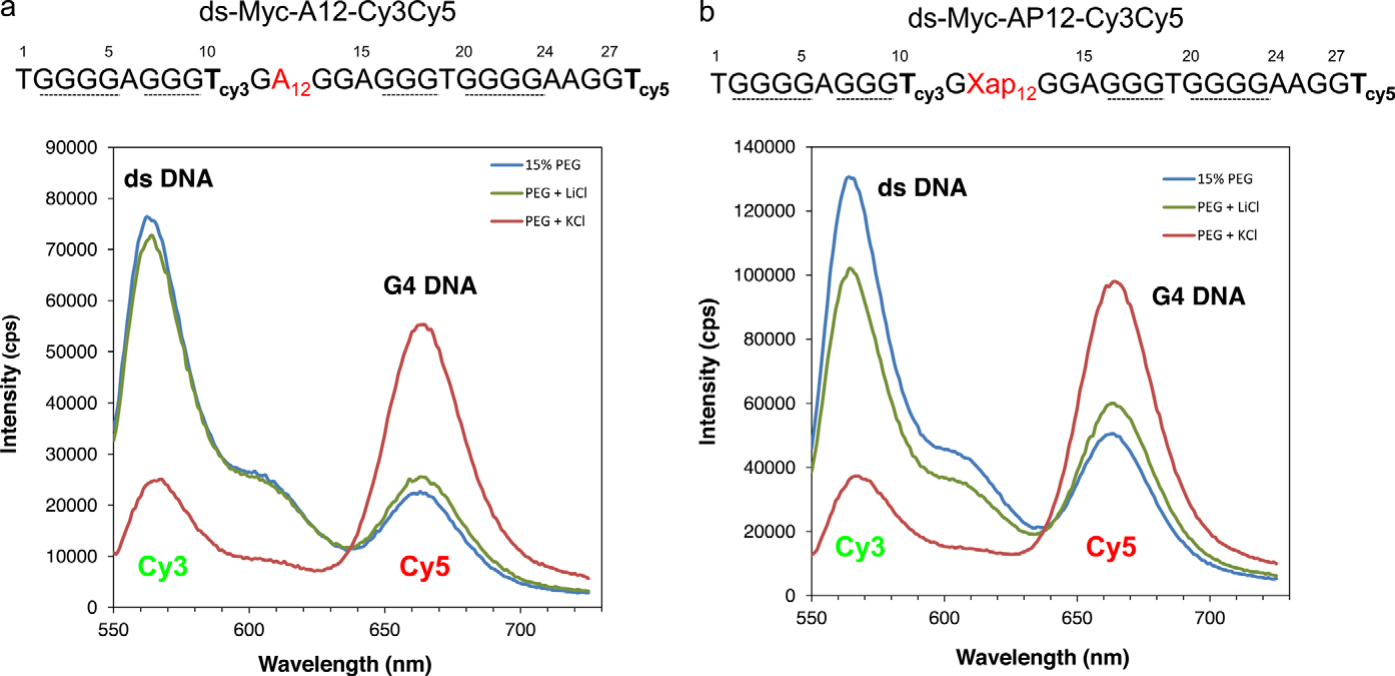
Detection of G4 DNA in ds c-myc-A12 or AP12 by FRET. (**a**) Emission spectra of ds myc-A12. (**b**) Emission spectra of ds myc-AP12. DNA substrates were annealed in 15% PEG 200 in the presence or absence of KCl as described in Materials and Methods. Control samples in which KCl was substituted with LiCl were run in parallel.

**Figure 7.**
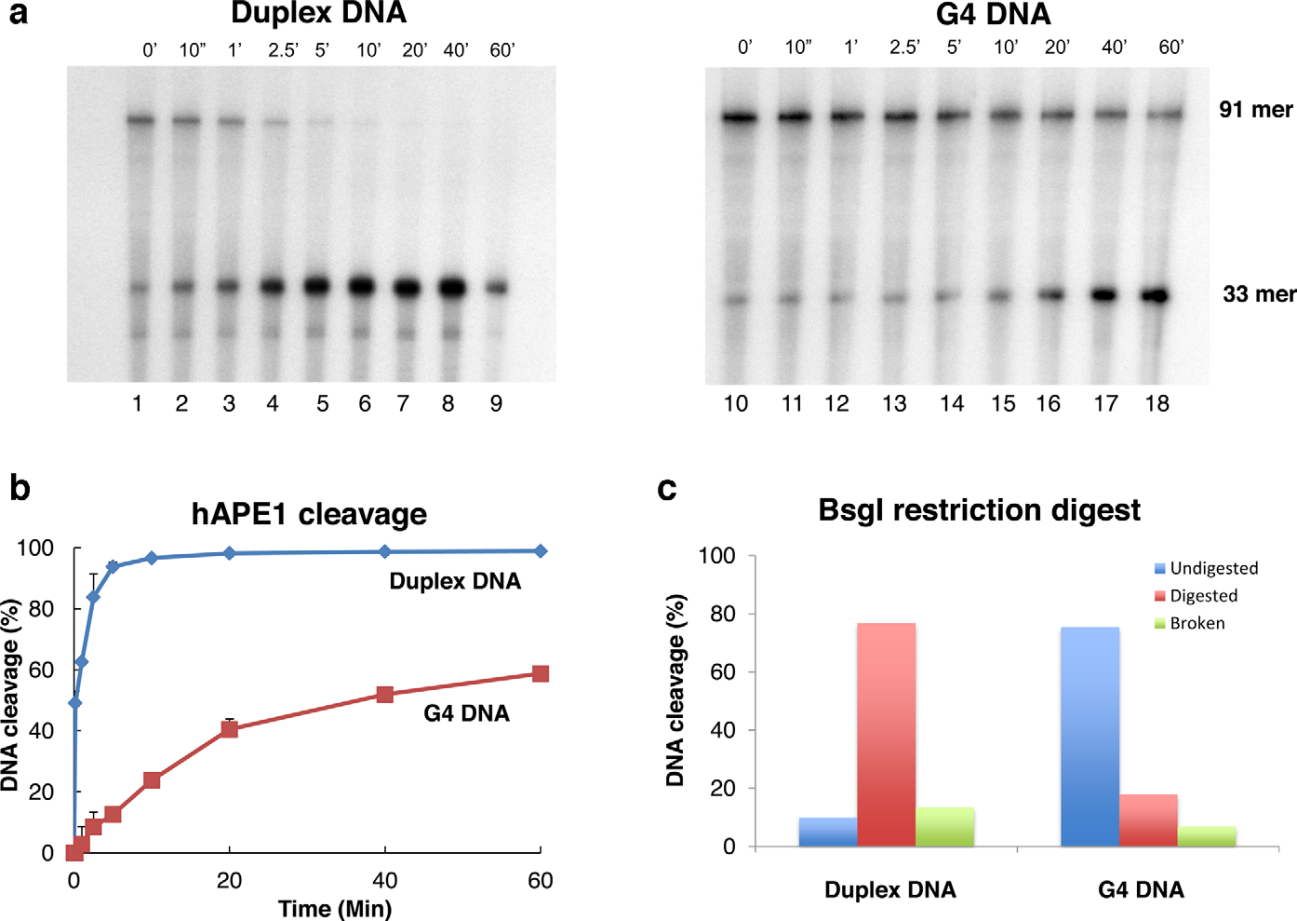
Slow kinetics of incision by hAPE1 of a single AP site located in quadruplex c-myc-AP12. (**A**) ds-c-myc-AP12 (91 mer) in duplex (left panel) or quadruplex (right panel) DNA conformation; 33 mer, 33 nt long APE1 cleavage product; time points are indicated above each gel picture. (**B**) Quantitation of the results of three independent hAPE1 experiments. (**C**) Quantitation of the extent of DNA in duplex or quadruplex structure by BsgI restriction digestion. Broken DNA, 33 mer product generated from cleavage at the AP site during heat denaturation; undigested DNA, 91 mer substrate resistant to BsgI cleavage.

### Electrophoretic mobility shift assays

A 91-mer oligonucleotide containing the G4-forming sequence and tetrahydrofuran at position 12 of the c-myc NHEIII_1_ was labeled at the 5' end with ^32^P and annealed to a complementary strand as described above. This substrate (40 nM) was mixed at room temperature with APE1-D210A (1.8 μM) in electrophoretic mobility shift assay buffer containing 50 mM HEPES pH 7.5, 10 mM NaCl, 10 mM MgCl_2_, 5% glycerol, 1 mM DTT, 1 mM EDTA, 0.1 mg/ml BSA. Ten millimolar KCl and 2% PEG were also included in control reaction mixtures containing duplex DNA to reproduce identical conditions as those of reactions containing the G4 substrate. Competitor DNA consisted of double-stranded DNA of identical sequence, in which the c-myc repeat was in G4 or B structure. Aliquots of the binding mixtures were chilled in ice after incubation for 10 min at room temperature. The protein-bound DNA was separated from the free substrate by electrophoresis on a 6% non-denaturing polyacrylamide gel at 8 V/cm^2^ for 120 min. Gels were dried and autoradiographed using intensifying screens.

### Fluorescence measurements

Fluorescence emission spectra were recorded using a QuantaMaster QM-1 spectrofluorometer equipped with excitation and emission monochromators and red-sensitive Hamamatsu R928 photomultiplier tubes (Photon Technology International). The Cy5 donor was excited at 540 nm and emission was measured from 550 to 725 nm using a 4-nm bandpass. DNA solutions were made by adding 4 μl of 1 μM DNA to 76 μl of buffer, as indicated in the figures, directly to a microcuvette.

## RESULTS

### Detection of G4 DNA structure in double-stranded c-myc-WT by DMS footprinting

A 91 mer oligonucleotide containing the c-myc NHEIII**_1_** (c-myc-WT, Table 1) was annealed to the complementary strand in the presence of 100 mM KCl and 20% PEG 200. PEG 200 is a molecular crowding agent that has been shown to facilitate the transition from duplex to quadruplex DNA by excluded volume effects, conditions mimicking the intracellular environment, which is characterized by high concentration of macromolecules (10–40% of the total cellular volume) and potassium ions (30–32). We directly tested the presence of G4 DNA in ds c-myc-WT by DMS footprinting experiments, a technique routinely used to identify G4 DNA formed in single-stranded synthetic oligonucleotides *in vitro* (25,33,34). This assay is based on the property of DMS to attack the N7 of guanine, which is accessible to DMS methylation when it is present in single-stranded or duplex DNA but not when it is paired to the exocyclic amino group of a neighboring guanine in a G-quartet (1,35). As a result, the Gs in the quartet are protected from methylation and subsequent piperidine cleavage compared to the duplex DNA control, resulting in a decreased intensity or absence of the corresponding G band after DNA separation on sequencing gels.

When ds c-myc-WT was treated with DMS and piperidine, followed by 12% denaturing polyacrylamide gel electrophoresis (Figure 1B, lane 1), bands of similar intensities corresponding to the Gs present in the G repeat and flanking sequences were observed. When c-myc-WT was annealed to the complementary strand in the presence of 100 mM KCl and 20% PEG 200 prior to DMS treatment, we observed decrease in intensity of several bands corresponding to Gs within the c-myc repeat (Figure 1, lane 2). Protection of G7-G9, G16-G18 and G20-G22 is expected from formation of either myc-1245 or myc-2345. Protection of G11-G14 is expected only if myc-2345 forms. Protection of G3-G5 is expected only if myc-1245 forms. Partial protection of G11-G14 and G3-G5 would be expected if both structures formed, and the relative levels of protection would depend on which structure predominated. We detected partial protection of G11 and G14. Protection of G3-G5, which only participate in myc-1245 but not in myc-2345 structure, was undetectable, suggesting that in the ds c-myc-WT the structure myc-2345 is predominant or more stable than myc-1245 during DMS treatment.

### Detection of G4 DNA structure in ds c-myc-WT by restriction digestion

As a quick diagnostic to confirm the presence of G4 DNA in our substrates and to obtain a more accurate estimate of the extent of the structural transition, we measured the fraction of DNA present in the G4 structure in the ds c-myc-WT by inhibition of DNA digestion by the restriction enzyme BsgI (36). BsgI cleaves the DNA 16 nt 3' from its recognition site in double-stranded B DNA but not when the DNA is in G4 (Figure 2). Consistent with G4 DNA formation in the majority of ds c-myc-WT, we found that BsgI cleavage was completely inhibited when the double-stranded DNA substrate, in which both strands contained a 5'-^32^P label, was pre-treated with PEG and KCl, to induce G4 DNA (Figure 2, lane 5). A PvuII restriction site is located upstream of the G4 sequence and should be in a duplex region regardless of whether a G4 structure is present. Digestion with restriction enzyme PvuII was complete under the same reaction conditions used for BsgI, confirming that inhibition of BsgI cleavage was due to structure formation (Figure 2, lane 4).

### Development of a FRET-based method for G4 detection in damaged or undamaged DNA

DMS protection assays together with BsgI restriction digestions indicated the presence of G4-DNA in ds c-myc-WT (Figures 1 and 2). However, DMS protection assays are not very informative to detect G4 formation in DNA substrates containing spontaneous DNA lesions, like abasic sites or 8-oxoguanine, due to their lability and resulting sensitivity to piperidine treatment (37). To verify structure formation in the damaged-containing substrate, we have utilized FRET-based assays (38). Based on our recent findings (24) showing that the presence of an AP site at position 12 of the c-myc repeat generated G4 structure myc-1245, we modified the DNA strand containing the G4 sequence by incorporating two fluorophores, Cy3 at position 10 and Cy5 at position 28 of the c-myc G repeat during oligonucleotide synthesis (Figure 3A and B). Based on the myc-1245 conformation, when the G repeat sequence exists in the B-DNA conformation, the donor Cy3 (D) is relatively far from the acceptor Cy5 (A) (about 60 Å assuming a rise of about 3.3 Å per base pair) and will not be efficiently quenched by the acceptor (Figure 3A and B). When the G4 DNA structure is formed, the donor Cy3 moves closer to the acceptor Cy5 and this decreases/quenches the donor fluorescence and increases the acceptor fluorescence due to resonance energy transfer (Figure 3A). First, we tested the FRET approach on a single-stranded DNA, ss c-myc-A12 substrate (Figure 3B, Table 1), which we had previously shown forms a stable c-myc-1245 G4 structure (24). As predicted, transition to G4 structure in the presence of KCl increased Cy5 acceptor emission compared to that measured for the untreated control. This indicated that FRET was an effective indicator of G4 DNA formation in the c-myc substrate. Next, we tested our FRET approach for G4 formation in the abasic site-containing ss c-myc-AP12 substrate. We found that pre-incubation of the AP site-containing substrate with KCl resulted in significant increase in Cy5 acceptor fluorescence (Figure 3C), indicating that G4 DNA formed in this substrate also. In agreement with the FRET data, we found that transcription of ss c-myc-A12 or ss c-myc-A12-Cy3-Cy5 pre-incubated in 100 mM KCl resulted in generation of 30 and 31 nt transcripts, as expected from formation of the myc-1245 structure (Figure 4, lanes 2 and 4) (24). Transcription of ss c-myc-A12-Cy3-Cy5 after incubation in the absence of 100 mM KCl resulted in formation of mostly a 39 nt RNA, as expected from transcription arrest at the Cy3 fluorophore at position 10. Under the same conditions ss c-myc-A12 generated a 74 nt RNA, as expected from transcription up to the end of ss c-myc-A12.

### Effect of PEG concentration on G4 detection in double-stranded DNA by FRET

Having established that our FRET approach efficiently detects G4 DNA formation in ss c-myc-A12 or ss c-myc-AP12 (Figure 3), we have extended our analyses to the corresponding double-stranded sequences. We have previously shown that the preparation of G4 DNA containing double-stranded substrates requires annealing of the complementary DNA strands in the presence of PEG and KCl (23) (Figure 1). To determine the optimal buffer conditions to measure FRET in double-stranded substrates, ss c-myc-A12 was annealed to the complementary strand in the presence or absence of 100 mM KCl and increasing concentrations of PEG 200 ranging from 0 to 40% (Figure 5). We observed a significant increase in Cy5 fluorescence compared to the duplex DNA control in the samples treated with PEG and KCl at PEG concentrations as low as 10% (Figure 5B). However, at PEG concentrations of 30% or greater, an increase in Cy5 acceptor fluorescence was also observed in the samples incubated in the absence of KCl. This suggests that intermolecular complexes may form between duplexes at high PEG concentrations that promote FRET without G4 formation (Figure 5A). Based on these data, we selected a concentration of 15% PEG with 100 mM KCl for our structural analyses on ds c-myc-AP12.

### Detection of G4 DNA in ds c-myc-AP12 by FRET

The DNA strand containing an abasic site or a base substitution G->A at position 12 of the c-myc G repeat was annealed to the complementary strand in the presence of 100 mM KCl and 15% PEG, followed by FRET analysis. Controls treated with PEG only or with PEG and 100 mM LiCl were annealed as well. Significant FRET was measured in the samples treated with PEG and KCl, as indicated by increased fluorescence of the Cy5 acceptor fluorophore compared to that measured for the samples treated with PEG only or with PEG and LiCl (Figure 6A and B). Based on these data, we concluded that G4 DNA was present in the double-stranded damaged and undamaged substrate.

### Slow incision at abasic sites located in G4 structures

Next, we have determined whether the presence of the abasic site in the G4 structure interfered with damage removal by repair enzymes. To this goal, we have compared the efficiency of AP site incision by hAPE1 on ds-c-myc-AP12 in duplex or quadruplex structure. Following incubation with hAPE1 for a defined period of time, DNA products were separated on denaturing polyacrylamide gels. To reproduce identical conditions as those of reactions containing the G4 substrate, 10 mM KCl and 1.5% PEG were also included in reaction mixtures containing duplex DNA (Figure 7A, lanes 1–9). In agreement with our hypothesis that lesions located at unusual DNA structures are poorly repaired by excision repair enzymes, we found that the rate of incision by hAPE1 was significantly slower when the AP site was located in the G4 structure compared to the duplex DNA control (Figure 7). BsgI digestion indicated that ∼70% of the substrate was in G4 when incubated with PEG and KCl (Figure 7C).

### Effect of G4 structure on binding of hAPE1 to ds c-myc-AP12

**Figure 8.**
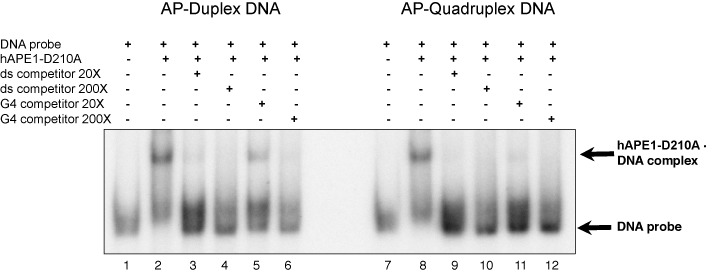
Binding of hAPE1 to an AP site in duplex or quadruplex DNA structure. Electrophoretic mobility shift assays were carried out to analyse binding of hAPE1 to DNA probes consisting of double-stranded substrates containing an AP site at position 12 of the c-myc G repeat. ds-c-myc-AP12 was incubated in the presence or absence of PEG and KCl to induce G4 formation, followed by incubation with hAPE1 in binding buffer at room temperature in the presence or absence or competitor DNA. Competitor DNA consisted of identical sequence in duplex or quadruplex DNA conformation. After incubation the binding reaction was immediately loaded on a 6% non-denaturing polyacrylamide gel.

Having established that the presence of the G4 structure interferes with hAPE1 incision, we carried out electrophoretic mobility shift experiments with hAPE1 to determine whether the observed decrease in enzymatic activity was due to decrease in binding to the lesion or to an inefficient chemical step of incision at the site of damage. To facilitate these analyses, we utilized a mutant form of hAPE1 (hAPE1-D210A) which has lost enzymatic activity but retains the DNA binding properties of the wt enzyme (39). When we compared hAPE1 binding to ds c-myc-AP12 in duplex versus quadruplex structure, we found that the enzyme bound to these DNA substrates with similar efficiency (Figure 8, lanes 2 and 8). When we challenged hAPE1 binding to the labeled DNA substrate with cold duplex or quadruplex competitor DNA, we found that both substrates efficiently competed with the labeled DNA, as indicated by a decrease in the amount of protein-DNA complex of 97% and 89%, respectively (Figure 8, lanes 3 and 5). These results indicate that the inefficient removal of a single AP site within a quadruplex structure is mostly the result of a decrease in enzymatic activity and not of a decrease in enzyme binding affinity to damaged DNA.

## DISCUSSION

We describe a FRET-based approach to monitor formation of G4 DNA structure in damage-containing DNA under molecular crowding conditions mimicking those found in cells (23,24). We have utilized this approach to directly show for the first time that the c-myc NHEIII_1_ G repeat folds into G4 structure when a single abasic site is located within the repeat. We further show that incision of the AP site by hAPE1 is significantly reduced by the presence of the G4 structure. And furthermore, that this effect is the consequence of a decrease in hAPE1 enzymatic activity and not of binding of the enzyme to G4 DNA.

The FRET-based approach takes advantage of the decrease in the distance between a donor Cy3 and an acceptor Cy5 fluorophore when they are located in G4 DNA compared to when they are in B form DNA (Figure 3A and B) (38,40). When c-myc-WT folds in G4 structure, two conformations, c-myc 1245 and the predominant c-myc 2345 are formed, depending on which G repeat is utilyzed (Figure 1 and (24)). We have shown previously that a single base substitution G12-> A12 is sufficent to shift the c-myc repeat from c-myc 2345 to c-myc-1245 structure (24). Similarly, we have found that a single abasic site located at position 12 of the c-myc repeat results in structure myc-1245 (24). Based on these observations, we reasoned that a decrease in the distance between the T10 and T28 from ∼60 Å (18 bp) when the c-mycA12 or the c-mycAP12 repeat is folded in B DNA to a much shorter distance when folded in G4 DNA would be sufficient to be detected by FRET. Consistent with our prediction, significant FRET was measured for the undamaged ss c-myc-A12 as well as the AP site-containing ss c-myc-AP12, as indicated by an increase in the emission peak corresponding to the acceptor (Cy5) fluorophore in the DNA preincubated with KCl and corresponding decrease of that of the donor (Cy3) fluorophore compared to the untreated control (Figure 3C and D). Our FRET data were confirmed by transcription stop assays, showing that pre-incubation in 100 mM KCl promoted formation of c-myc1245 structure, as indicated by synthesis of 30 and 31 nt transcripts after transcription of ss c-myc-A12-cy3-cy5 by T7 RNA polymerase (Figure 4 and (24)).

To facilitate G4 structure formation in the double-stranded substrate, in which the transition from B to G4 DNA is energetically disfavored, DNA substrates were annealed in the presence of a crowding agent, PEG, in addition to potassium. These conditions have been described as mimicking those found in living cells (30,31,41). They were previously utilized in our laboratory to show G4 DNA formation in the double-stranded G repeat from the c-myb promoter (23). In the presence of PEG, the transition to G4 DNA was detected by FRET at concentrations as low as 10% (Figures 6 and 7). In addition, in support of G4 formation in the abasic site containing substrates, strong FRET was detected only in the sample treated with PEG and KCl and undetectable FRET signal was measured when KCl was substituted with LiCl (Figure 6A and B). PEG concentrations of 30% or greater resulted in significant FRET measured in the DNA samples independent of prior treatment with KCl (Figure 5A). This suggests that intermolecular complexes may form between duplexes at high PEG concentrations that promote FRET without G4 formation.

Based on our FRET data, we conclude that the c-myc NHEIII_1_ G repeat containing an AP site at position 12 folds in G4 in single- and double -stranded DNA. This is the first report showing G4 structure formation in double-stranded damage-containing DNA. G4 structure formation was recently described in single-stranded AP or 8-oxoG containing human telomere and in AP site containing-d(TGGGGdspacerT) (42–44). Our method is reliable and effective to detect G4 DNA formation in damage-containing DNA. It has the advantage over existing enzymatic (DNA polymerase, RNA polymerase stop assays), electrophoretic, chemical and biophysical assays (DMS footprinting, circular dichroism, thermal stability curves) (reviewed in (45)) to allow the direct measurement of the formation of G4-DNA in solution. In addition, because it does not require the use of chemical or enzymatic reagents, the data obtained represent a faithful representation of the likely structure formed during the repair reaction.

Having established that G4 DNA forms in ds c-myc-AP12, we have investigated whether the presence of the G4 structure interferes with incision of the AP site by hAPE1 (Figure 7). Slower kinetics of hAPE1 incision were observed in G4 DNA compared to B DNA, with 50% AP site incision observed after 40 min incubation for the G4 containing substrate compared to 10 s required to cleave the duplex DNA substrate of identical sequence. This indicated that hAPE1 activity was ∼200 times faster when an AP site was located in B DNA than when it was in G4 DNA. During incubation with enzyme ∼70% of our substrate was in G4 structure as indicated by inhibition of BsgI restriction enzyme digestion (Figure 7). In addition, band shift experiments revealed that hAPE1 bound with similar affinity to G4 and B DNA, suggesting that enzyme catalytic activity was the limiting step in completing DNA cleavage at the AP site (Figure 8).

The unusual features of the double-stranded substrate with respect to the canonical B DNA may explain the observed slower rate of damage removal by hAPE1. Available crystal structures of hAPE1 bound to double-stranded AP DNA reveal that the enzyme recognizes the presence of an AP site by contacting not only the AP containing DNA strand trough binding of few nucleotides around the lesion, but also by making contacts with the opposite strand (46,47). Significant bending of the DNA is necessary for the enzyme to fit the AP DNA into a narrow pocket before catalysis can occur. These structural requirements are lacking in the G4 substrate, likely explaining the poor activity measured in G4 DNA compared to B DNA. In agreement with this possibility, it has been shown that hAPE1 cleaves DNA at AP sites less efficiently when these lesions are in single-stranded DNA compared to their double-stranded counterpart (48,49). In addition, repair of 8-oxoG by human OGG1 located in the loop of a hairpin substrate occurs with a rate that is ∼700 fold slower than that observed for the duplex substrate (18).

We conclude that we have developed an effective *in vitro* system for monitoring G4 structure formation in damage-containing DNA, thus providing an effective tool to investigate the mechanistic details of repair occurring at this non-canonical DNA structure. By characterizing the mechanistic details of how the presence of this structure may promote (i) erroneous recruitment of DNA-processing enzymes at or near these regions and (ii) inefficient and faulty repair of DNA lesions, we expect to increase our understanding of how mutations are generated at high frequency at these sites.
